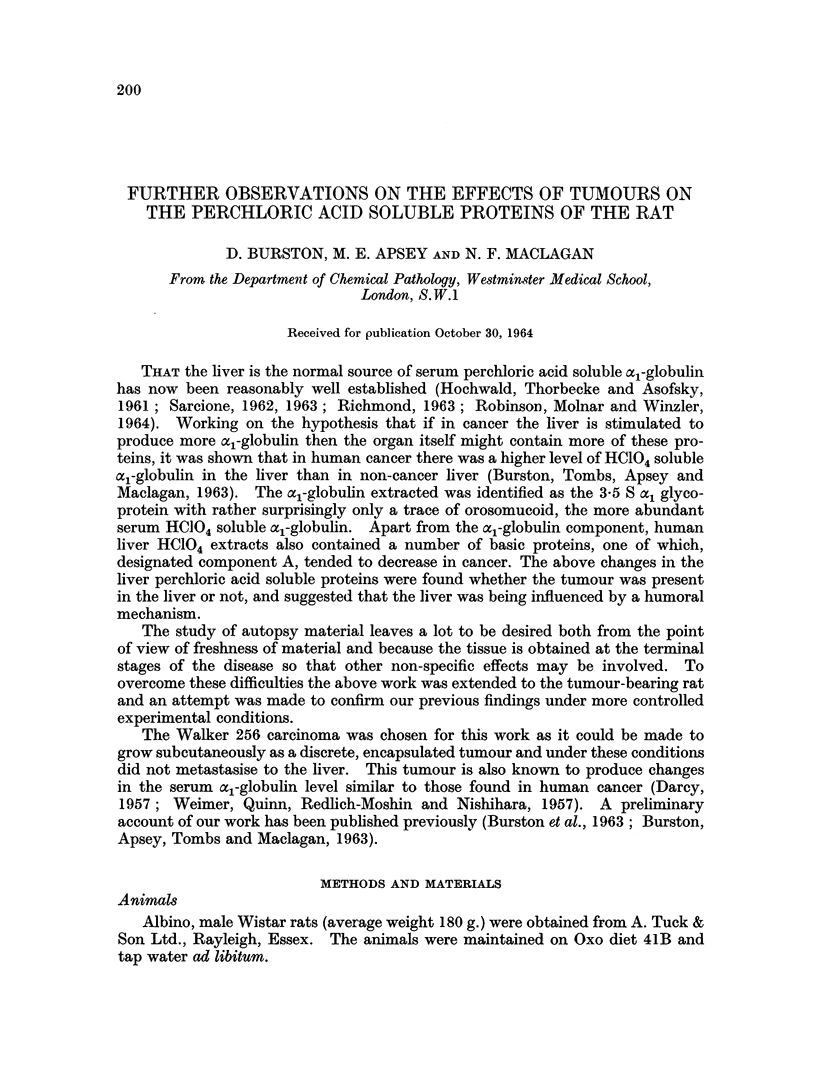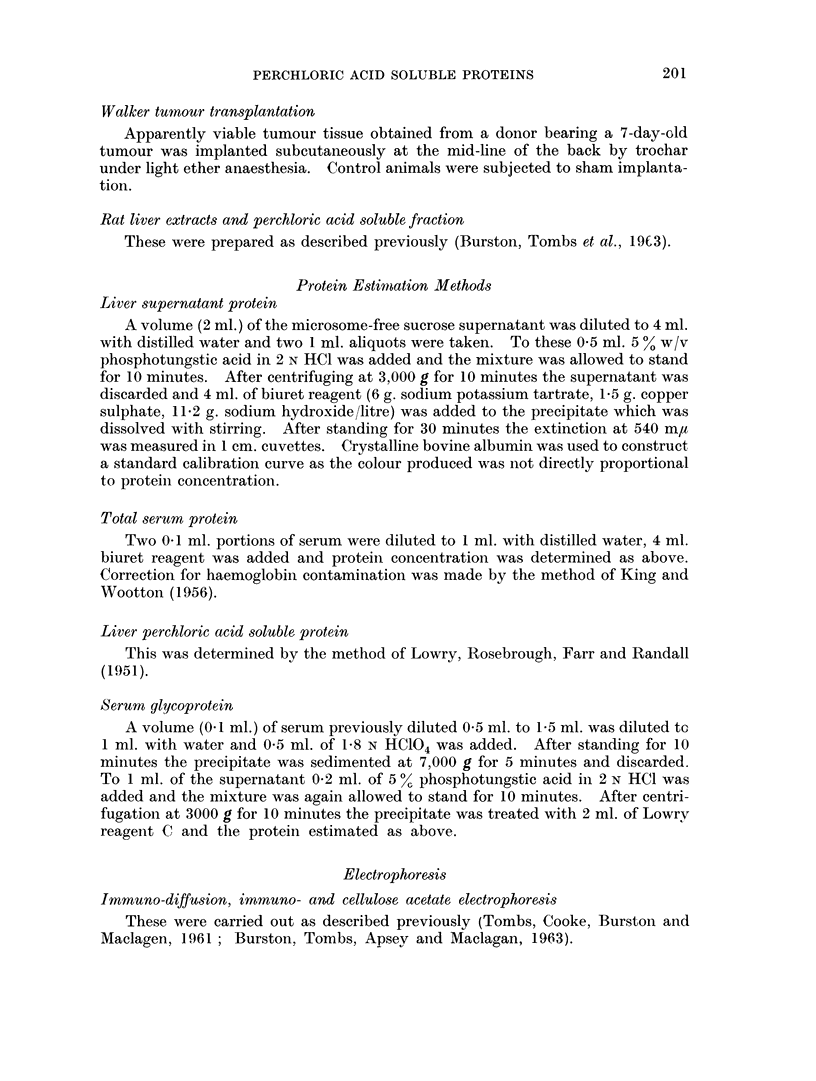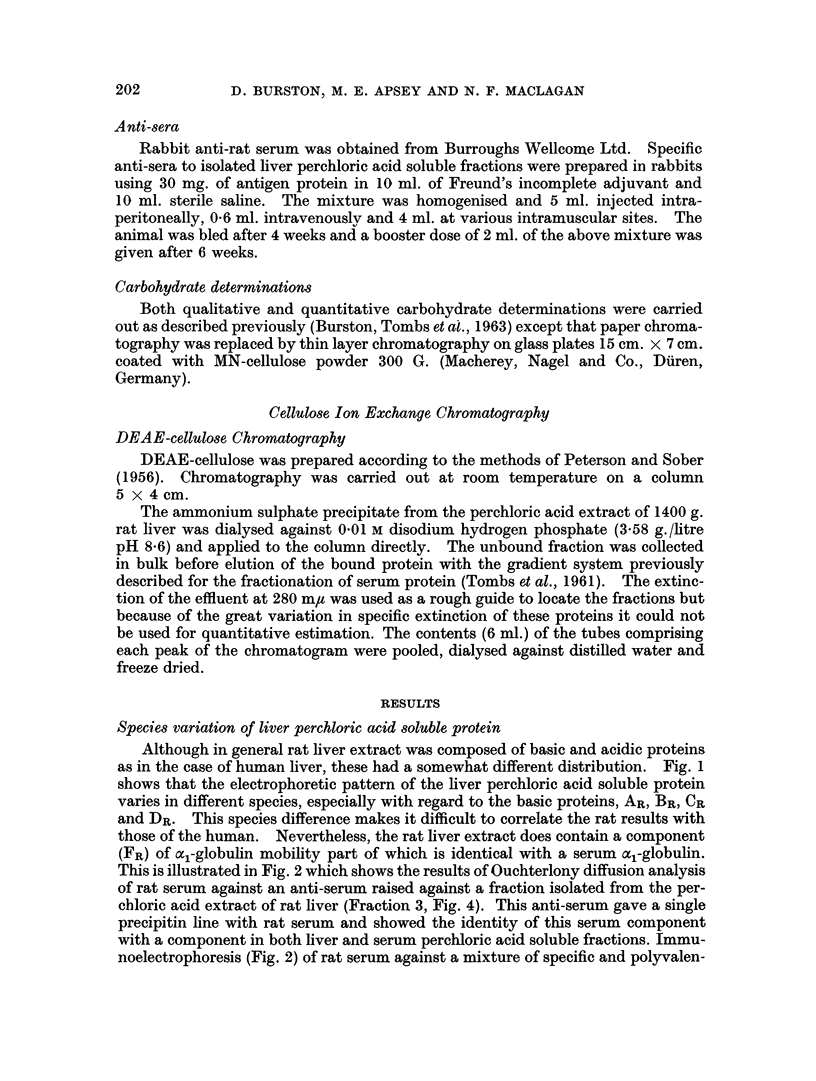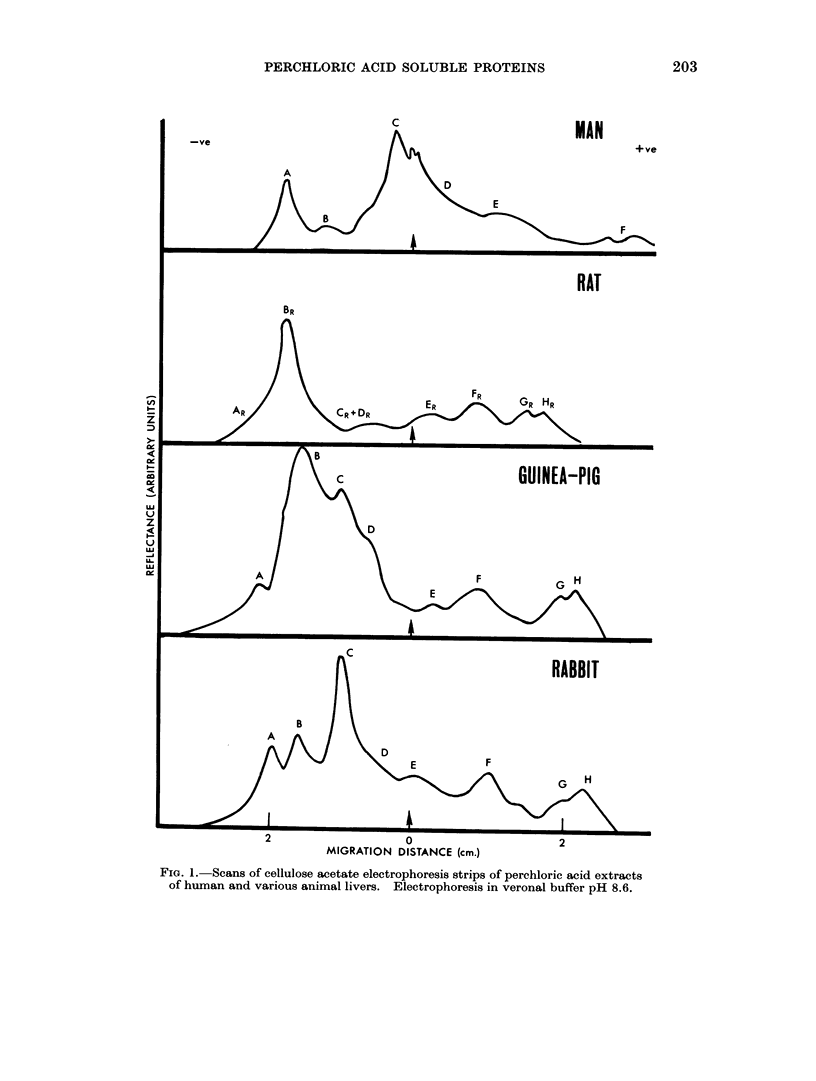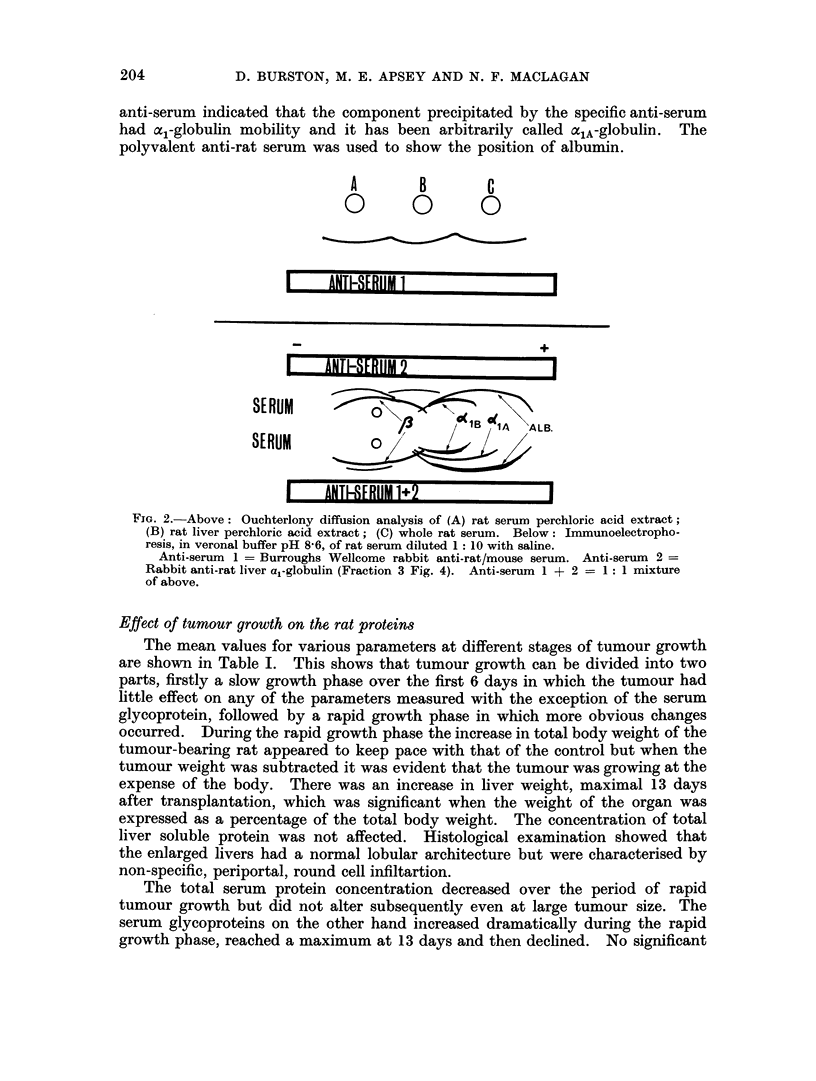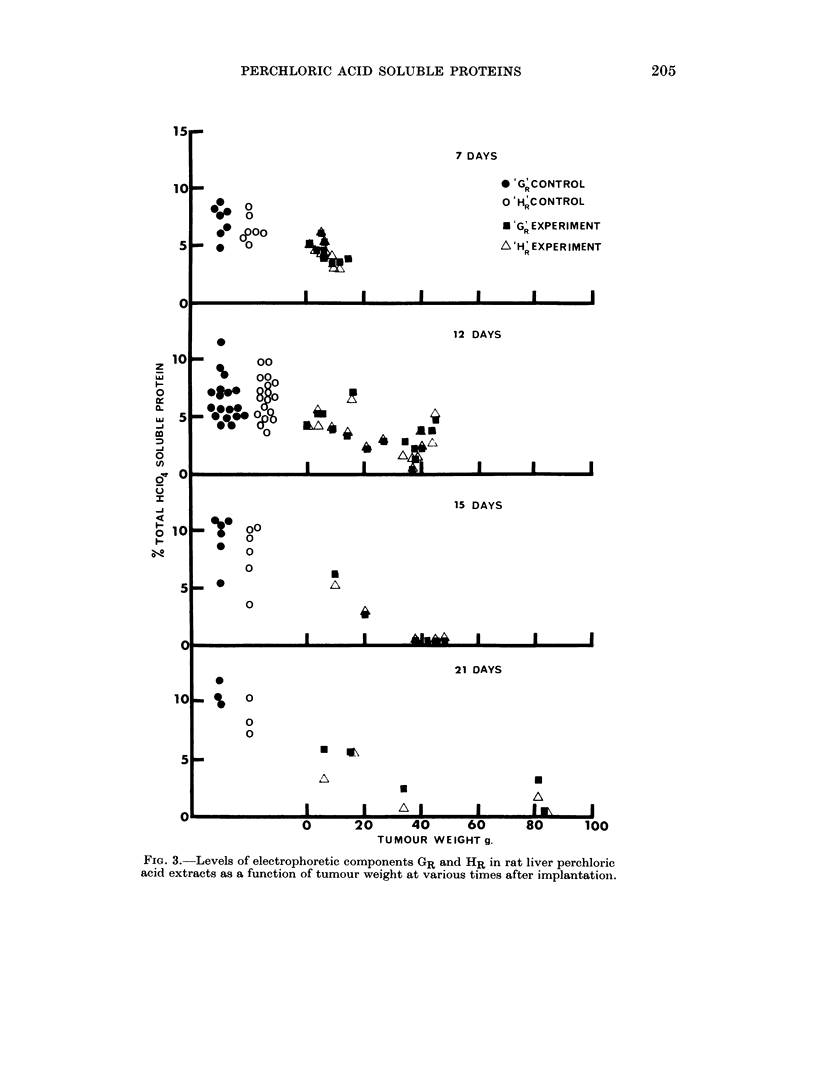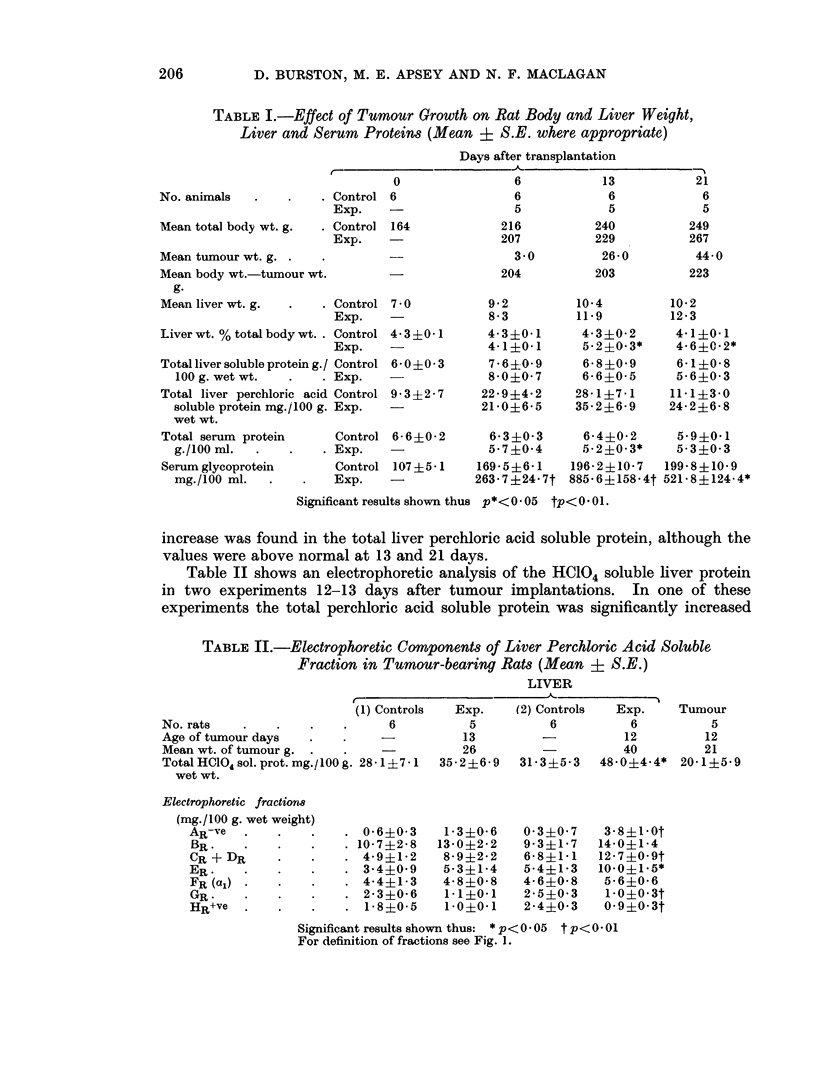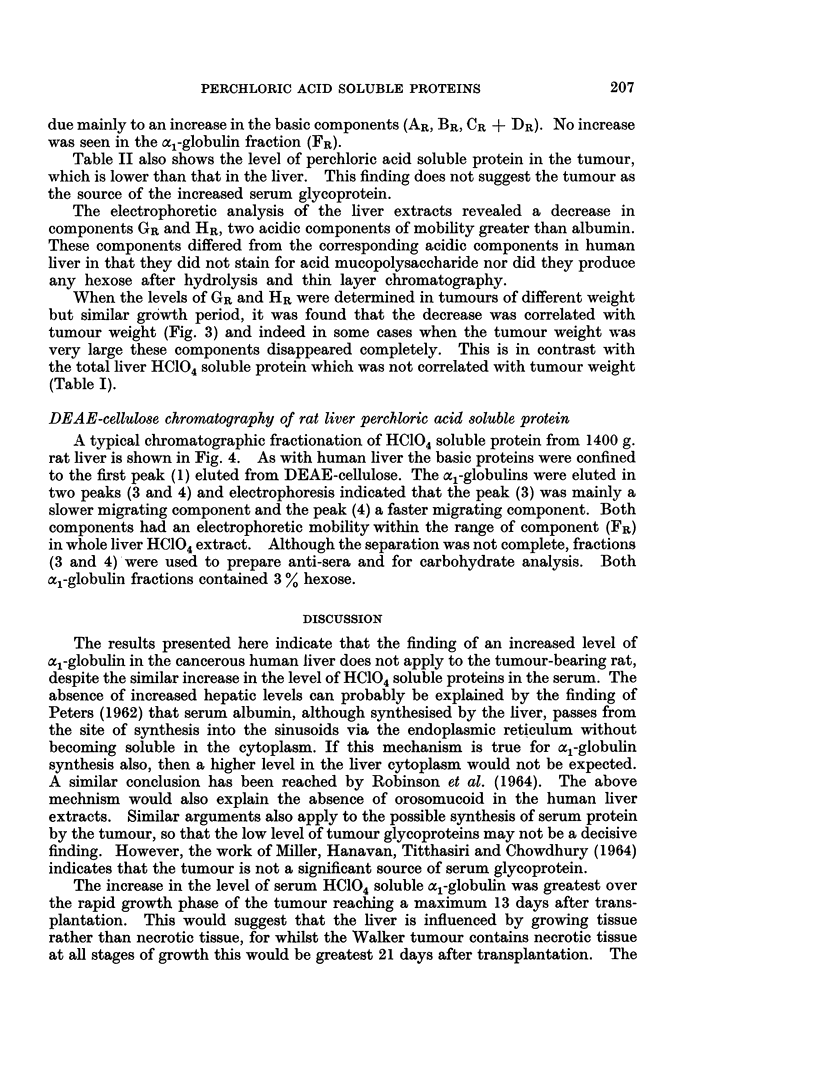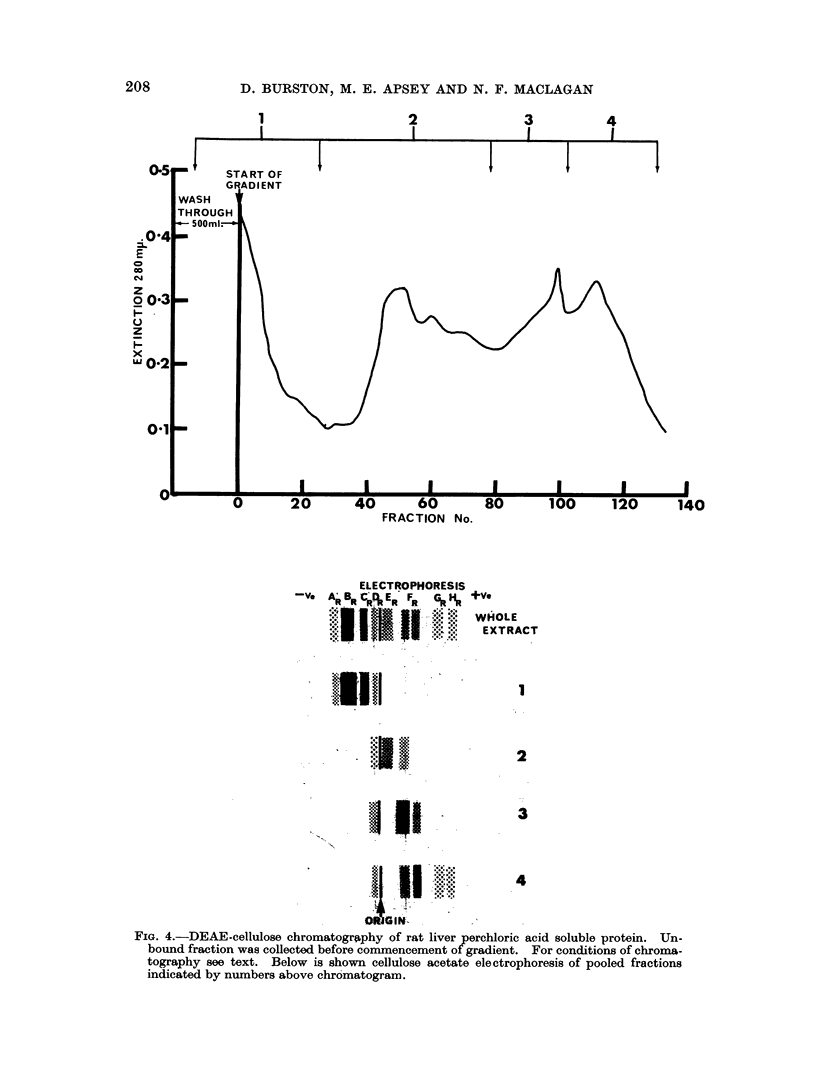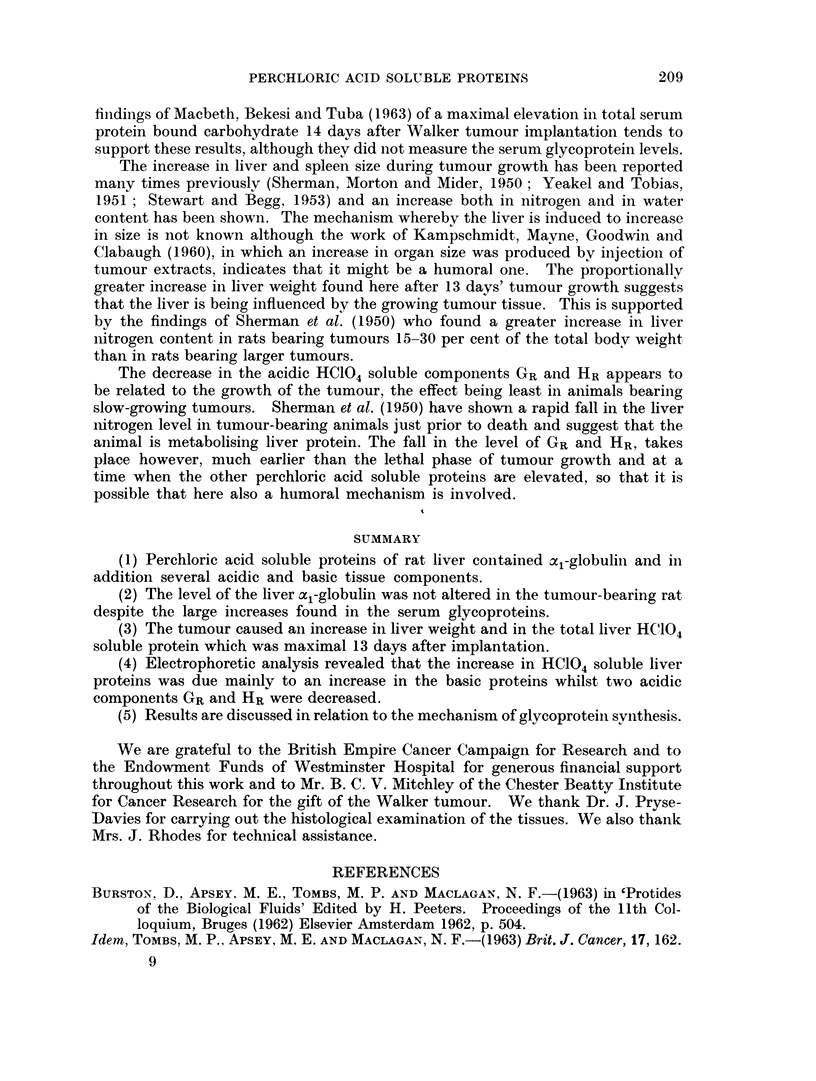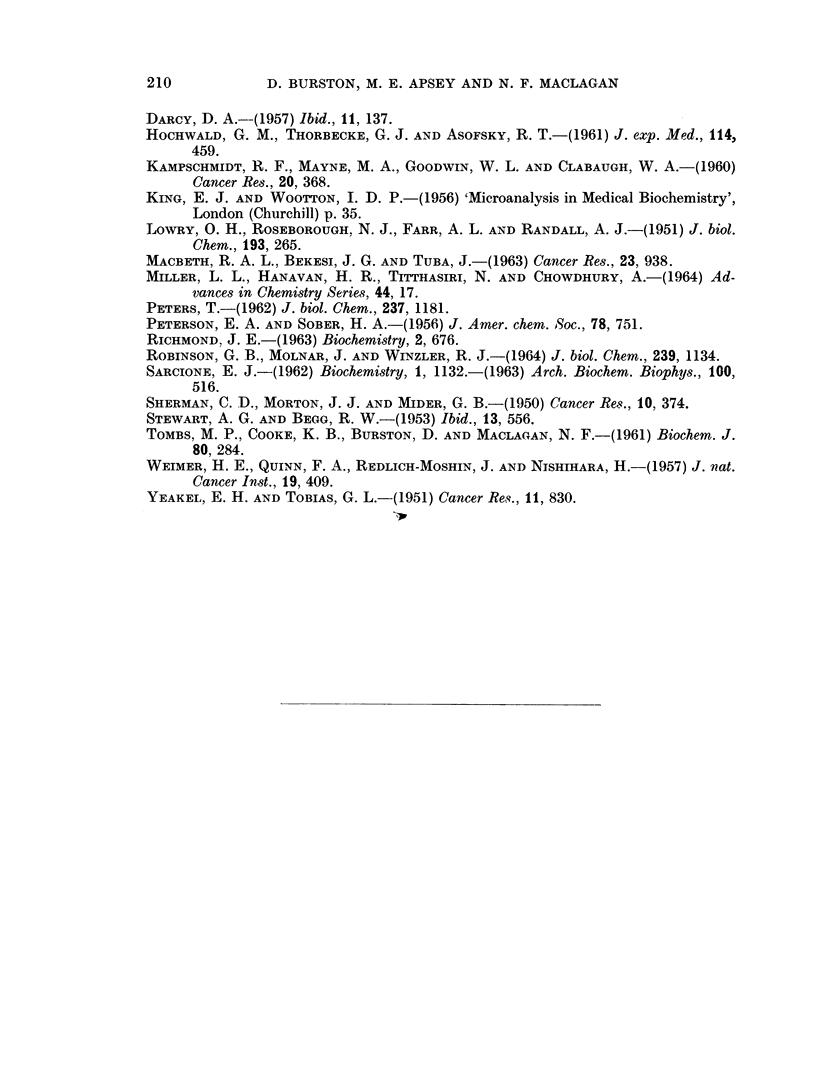# Further Observations on the Effects of Tumours on the Perchloric Acid Soluble Proteins of the Rat

**DOI:** 10.1038/bjc.1965.25

**Published:** 1965-03

**Authors:** D. Burston, M. E. Apsey, N. F. Maclagan


### FURTHER OBSERVATIONS ON THE EFFECTS OF TUMOURS ON THE PERCHLORIC ACID SOLUBLE PROTEINS OF THE RAT

D. BURSTON, M. E. APSEY AND N. F. MACLAGAN

*From the Department of Chemical Pathology, Westminster Medical School, London, S.W.1*

Received for publication October 30, 1964

THAT the liver is the normal source of serum perchloric acid soluble $\alpha_1$-globulin has now been reasonably well established (Hochwald, Thorbecke and Asofsky, 1961 ; Sarcione, 1962, 1963 ; Richmond, 1963 ; Robinson, Molnar and Winzler, 1964). Working on the hypothesis that if in cancer the liver is stimulated to produce more $\alpha_1$-globulin then the organ itself might contain more of these proteins, it was shown that in human cancer there was a higher level of $HClO_4$ soluble $\alpha_1$-globulin in the liver than in non-cancer liver (Burston, Tombs, Apsey and Maclagan, 1963). The $\alpha_1$-globulin extracted was identified as the 3·5 S $\alpha_1$ glycoprotein with rather surprisingly only a trace of orosomucoid, the more abundant serum $HClO_4$ soluble $\alpha_1$-globulin. Apart from the $\alpha_1$-globulin component, human liver $HClO_4$ extracts also contained a number of basic proteins, one of which, designated component A, tended to decrease in cancer. The above changes in the liver perchloric acid soluble proteins were found whether the tumour was present in the liver or not, and suggested that the liver was being influenced by a humoral mechanism.

The study of autopsy material leaves a lot to be desired both from the point of view of freshness of material and because the tissue is obtained at the terminal stages of the disease so that other non-specific effects may be involved. To overcome these difficulties the above work was extended to the tumour-bearing rat and an attempt was made to confirm our previous findings under more controlled experimental conditions.

The Walker 256 carcinoma was chosen for this work as it could be made to grow subcutaneously as a discrete, encapsulated tumour and under these conditions did not metastasise to the liver. This tumour is also known to produce changes in the serum $\alpha_1$-globulin level similar to those found in human cancer (Darcy, 1957 ; Weimer, Quinn, Redlich-Moshin and Nishihara, 1957). A preliminary account of our work has been published previously (Burston *et al.*, 1963 ; Burston, Apsey, Tombs and Maclagan, 1963).

### METHODS AND MATERIALS

### *Animals*

Albino, male Wistar rats (average weight 180 g.) were obtained from A. Tuck & Son Ltd., Rayleigh, Essex. The animals were maintained on Oxo diet 41B and tap water *ad libitum*.

*Walker tumour transplantation*

Apparently viable tumour tissue obtained from a donor bearing a 7-day-old tumour was implanted subcutaneously at the mid-line of the back by trochar under light ether anaesthesia. Control animals were subjected to sham implantation.

*Rat liver extracts and perchloric acid soluble fraction*

These were prepared as described previously (Burston, Tombs *et al.*, 1963).

### *Protein Estimation Methods*

*Liver supernatant protein*

A volume (2 ml.) of the microsome-free sucrose supernatant was diluted to 4 ml. with distilled water and two 1 ml. aliquots were taken. To these 0·5 ml. 5 % w/v phosphotungstic acid in 2 N HCl was added and the mixture was allowed to stand for 10 minutes. After centrifuging at 3,000 *g* for 10 minutes the supernatant was discarded and 4 ml. of biuret reagent (6 g. sodium potassium tartrate, 1·5 g. copper sulphate, 11·2 g. sodium hydroxide/litre) was added to the precipitate which was dissolved with stirring. After standing for 30 minutes the extinction at 540 m$\mu$ was measured in 1 cm. cuvettes. Crystalline bovine albumin was used to construct a standard calibration curve as the colour produced was not directly proportional to protein concentration.

*Total serum protein*

Two 0·1 ml. portions of serum were diluted to 1 ml. with distilled water, 4 ml. biuret reagent was added and protein concentration was determined as above. Correction for haemoglobin contamination was made by the method of King and Wootton (1956).

*Liver perchloric acid soluble protein*

This was determined by the method of Lowry, Rosebrough, Farr and Randall (1951).

*Serum glycoprotein*

A volume (0·1 ml.) of serum previously diluted 0·5 ml. to 1·5 ml. was diluted to 1 ml. with water and 0·5 ml. of 1·8 N $HClO_4$ was added. After standing for 10 minutes the precipitate was sedimented at 7,000 *g* for 5 minutes and discarded. To 1 ml. of the supernatant 0·2 ml. of 5 % phosphotungstic acid in 2 N HCl was added and the mixture was again allowed to stand for 10 minutes. After centrifugation at 3000 *g* for 10 minutes the precipitate was treated with 2 ml. of Lowry reagent C and the protein estimated as above.

### *Electrophoresis*

*Immuno-diffusion, immuno- and cellulose acetate electrophoresis*

These were carried out as described previously (Tombs, Cooke, Burston and Maclagen, 1961 ; Burston, Tombs, Apsey and Maclagan, 1963).

*Anti-sera*

Rabbit anti-rat serum was obtained from Burroughs Wellcome Ltd. Specific anti-sera to isolated liver perchloric acid soluble fractions were prepared in rabbits using 30 mg. of antigen protein in 10 ml. of Freund's incomplete adjuvant and 10 ml. sterile saline. The mixture was homogenised and 5 ml. injected intraperitoneally, 0·6 ml. intravenously and 4 ml. at various intramuscular sites. The animal was bled after 4 weeks and a booster dose of 2 ml. of the above mixture was given after 6 weeks.

*Carbohydrate determinations*

Both qualitative and quantitative carbohydrate determinations were carried out as described previously (Burston, Tombs *et al.*, 1963) except that paper chromatography was replaced by thin layer chromatography on glass plates 15 cm. × 7 cm. coated with MN-cellulose powder 300 G. (Macherey, Nagel and Co., Düren, Germany).

*Cellulose Ion Exchange Chromatography*

*DEAE-cellulose Chromatography*

DEAE-cellulose was prepared according to the methods of Peterson and Sober (1956). Chromatography was carried out at room temperature on a column 5 × 4 cm.

The ammonium sulphate precipitate from the perchloric acid extract of 1400 g. rat liver was dialysed against 0·01 M disodium hydrogen phosphate (3·58 g./litre pH 8·6) and applied to the column directly. The unbound fraction was collected in bulk before elution of the bound protein with the gradient system previously described for the fractionation of serum protein (Tombs *et al.*, 1961). The extinction of the effluent at 280 m$\mu$ was used as a rough guide to locate the fractions but because of the great variation in specific extinction of these proteins it could not be used for quantitative estimation. The contents (6 ml.) of the tubes comprising each peak of the chromatogram were pooled, dialysed against distilled water and freeze dried.

### RESULTS

*Species variation of liver perchloric acid soluble protein*

Although in general rat liver extract was composed of basic and acidic proteins as in the case of human liver, these had a somewhat different distribution. Fig. 1 shows that the electrophoretic pattern of the liver perchloric acid soluble protein varies in different species, especially with regard to the basic proteins, $A_R$, $B_R$, $C_R$ and $D_R$. This species difference makes it difficult to correlate the rat results with those of the human. Nevertheless, the rat liver extract does contain a component ($F_R$) of $\alpha_1$-globulin mobility part of which is identical with a serum $\alpha_1$-globulin. This is illustrated in Fig. 2 which shows the results of Ouchterlony diffusion analysis of rat serum against an anti-serum raised against a fraction isolated from the perchloric acid extract of rat liver (Fraction 3, Fig. 4). This anti-serum gave a single precipitin line with rat serum and showed the identity of this serum component with a component in both liver and serum perchloric acid soluble fractions. Immunoelectrophoresis (Fig. 2) of rat serum against a mixture of specific and polyvalen-

FIG. 1.—Scans of cellulose acetate electrophoresis strips of perchloric acid extracts of human and various animal livers. Electrophoresis in veronal buffer pH 8.6.

anti-serum indicated that the component precipitated by the specific anti-serum had $\alpha_1$-globulin mobility and it has been arbitrarily called $\alpha_{1A}$-globulin. The polyvalent anti-rat serum was used to show the position of albumin.

FIG. 2.—Above: Ouchterlony diffusion analysis of (A) rat serum perchloric acid extract; (B) rat liver perchloric acid extract; (C) whole rat serum. Below: Immunoelectrophoresis, in veronal buffer pH 8·6, of rat serum diluted 1 : 10 with saline.

Anti-serum 1 = Burroughs Wellcome rabbit anti-rat/mouse serum. Anti-serum 2 = Rabbit anti-rat liver $\alpha_1$-globulin (Fraction 3 Fig. 4). Anti-serum 1 + 2 = 1 : 1 mixture of above.

*Effect of tumour growth on the rat proteins*

The mean values for various parameters at different stages of tumour growth are shown in Table I. This shows that tumour growth can be divided into two parts, firstly a slow growth phase over the first 6 days in which the tumour had little effect on any of the parameters measured with the exception of the serum glycoprotein, followed by a rapid growth phase in which more obvious changes occurred. During the rapid growth phase the increase in total body weight of the tumour-bearing rat appeared to keep pace with that of the control but when the tumour weight was subtracted it was evident that the tumour was growing at the expense of the body. There was an increase in liver weight, maximal 13 days after transplantation, which was significant when the weight of the organ was expressed as a percentage of the total body weight. The concentration of total liver soluble protein was not affected. Histological examination showed that the enlarged livers had a normal lobular architecture but were characterised by non-specific, periportal, round cell infiltartion.

The total serum protein concentration decreased over the period of rapid tumour growth but did not alter subsequently even at large tumour size. The serum glycoproteins on the other hand increased dramatically during the rapid growth phase, reached a maximum at 13 days and then declined. No significant

FIG. 3.—Levels of electrophoretic components $G_R$ and $H_R$ in rat liver perchloric acid extracts as a function of tumour weight at various times after implantation.

TABLE I.—*Effect of Tumour Growth on Rat Body and Liver Weight, Liver and Serum Proteins (Mean ± S.E. where appropriate)*

| | | Days after transplantation | | | |
|---|---|---|---|---|---|
| | | 0 | 6 | 13 | 21 |
| No. animals | Control | 6 | 6 | 6 | 6 |
| | Exp. | — | 5 | 5 | 5 |
| Mean total body wt. g. | Control | 164 | 216 | 240 | 249 |
| | Exp. | — | 207 | 229 | 267 |
| Mean tumour wt. g. | | — | 3·0 | 26·0 | 44·0 |
| Mean body wt.—tumour wt. g. | | — | 204 | 203 | 223 |
| Mean liver wt. g. | Control | 7·0 | 9·2 | 10·4 | 10·2 |
| | Exp. | — | 8·3 | 11·9 | 12·3 |
| Liver wt. % total body wt. | Control | 4·3±0·1 | 4·3±0·1 | 4·3±0·2 | 4·1±0·1 |
| | Exp. | — | 4·1±0·1 | 5·2±0·3* | 4·6±0·2* |
| Total liver soluble protein g./100 g. wet wt. | Control | 6·0±0·3 | 7·6±0·9 | 6·8±0·9 | 6·1±0·8 |
| | Exp. | — | 8·0±0·7 | 6·6±0·5 | 5·6±0·3 |
| Total liver perchloric acid soluble protein mg./100 g. wet wt. | Control | 9·3±2·7 | 22·9±4·2 | 28·1±7·1 | 11·1±3·0 |
| | Exp. | — | 21·0±6·5 | 35·2±6·9 | 24·2±6·8 |
| Total serum protein g./100 ml. | Control | 6·6±0·2 | 6·3±0·3 | 6·4±0·2 | 5·9±0·1 |
| | Exp. | — | 5·7±0·4 | 5·2±0·3* | 5·3±0·3 |
| Serum glycoprotein mg./100 ml. | Control | 107±5·1 | 169·5±6·1 | 196·2±10·7 | 199·8±10·9 |
| | Exp. | — | 263·7±24·7† | 885·6±158·4† | 521·8±124·4* |

Significant results shown thus $p^* < 0·05$ $\dagger p < 0·01$.

increase was found in the total liver perchloric acid soluble protein, although the values were above normal at 13 and 21 days.

Table II shows an electrophoretic analysis of the $HClO_4$ soluble liver protein in two experiments 12–13 days after tumour implantations. In one of these experiments the total perchloric acid soluble protein was significantly increased

TABLE II.—*Electrophoretic Components of Liver Perchloric Acid Soluble Fraction in Tumour-bearing Rats (Mean ± S.E.)*

| | LIVER | | | | |
|---|---|---|---|---|---|
| | (1) Controls | Exp. | (2) Controls | Exp. | Tumour |
| No. rats | 6 | 5 | 6 | 6 | 5 |
| Age of tumour days | — | 13 | — | 12 | 12 |
| Mean wt. of tumour g. | — | 26 | — | 40 | 21 |
| Total $HClO_4$ sol. prot. mg./100 g. wet wt. | 28·1±7·1 | 35·2±6·9 | 31·3±5·3 | 48·0±4·4* | 20·1±5·9 |
| *Electrophoretic fractions* (mg./100 g. wet weight) | | | | | |
| $A_R$-ve | 0·6±0·3 | 1·3±0·6 | 0·3±0·7 | 3·8±1·0† | |
| $B_R$ | 10·7±2·8 | 13·0±2·2 | 9·3±1·7 | 14·0±1·4 | |
| $C_R + D_R$ | 4·9±1·2 | 8·9±2·2 | 6·8±1·1 | 12·7±0·9† | |
| $E_R$ | 3·4±0·9 | 5·3±1·4 | 5·4±1·3 | 10·0±1·5* | |
| $F_R$ ($\alpha_1$) | 4·4±1·3 | 4·8±0·8 | 4·6±0·8 | 5·6±0·6 | |
| $G_R$ | 2·3±0·6 | 1·1±0·1 | 2·5±0·3 | 1·0±0·3† | |
| $H_R$+ve | 1·8±0·5 | 1·0±0·1 | 2·4±0·3 | 0·9±0·3† | |

Significant results shown thus: $*p < 0·05$ $\dagger p < 0·01$
For definition of fractions see Fig. 1.

due mainly to an increase in the basic components ($A_R$, $B_R$, $C_R + D_R$). No increase was seen in the $\alpha_1$-globulin fraction ($F_R$).

Table II also shows the level of perchloric acid soluble protein in the tumour, which is lower than that in the liver. This finding does not suggest the tumour as the source of the increased serum glycoprotein.

The electrophoretic analysis of the liver extracts revealed a decrease in components $G_R$ and $H_R$, two acidic components of mobility greater than albumin. These components differed from the corresponding acidic components in human liver in that they did not stain for acid mucopolysaccharide nor did they produce any hexose after hydrolysis and thin layer chromatography.

When the levels of $G_R$ and $H_R$ were determined in tumours of different weight but similar growth period, it was found that the decrease was correlated with tumour weight (Fig. 3) and indeed in some cases when the tumour weight was very large these components disappeared completely. This is in contrast with the total liver $HClO_4$ soluble protein which was not correlated with tumour weight (Table I).

*DEAE-cellulose chromatography of rat liver perchloric acid soluble protein*

A typical chromatographic fractionation of $HClO_4$ soluble protein from 1400 g. rat liver is shown in Fig. 4. As with human liver the basic proteins were confined to the first peak (1) eluted from DEAE-cellulose. The $\alpha_1$-globulins were eluted in two peaks (3 and 4) and electrophoresis indicated that the peak (3) was mainly a slower migrating component and the peak (4) a faster migrating component. Both components had an electrophoretic mobility within the range of component ($F_R$) in whole liver $HClO_4$ extract. Although the separation was not complete, fractions (3 and 4) were used to prepare anti-sera and for carbohydrate analysis. Both $\alpha_1$-globulin fractions contained 3 % hexose.

### DISCUSSION

The results presented here indicate that the finding of an increased level of $\alpha_1$-globulin in the cancerous human liver does not apply to the tumour-bearing rat, despite the similar increase in the level of $HClO_4$ soluble proteins in the serum. The absence of increased hepatic levels can probably be explained by the finding of Peters (1962) that serum albumin, although synthesised by the liver, passes from the site of synthesis into the sinusoids via the endoplasmic reticulum without becoming soluble in the cytoplasm. If this mechanism is true for $\alpha_1$-globulin synthesis also, then a higher level in the liver cytoplasm would not be expected. A similar conclusion has been reached by Robinson *et al.* (1964). The above mechnism would also explain the absence of orosomucoid in the human liver extracts. Similar arguments also apply to the possible synthesis of serum protein by the tumour, so that the low level of tumour glycoproteins may not be a decisive finding. However, the work of Miller, Hanavan, Titthasiri and Chowdhury (1964) indicates that the tumour is not a significant source of serum glycoprotein.

The increase in the level of serum $HClO_4$ soluble $\alpha_1$-globulin was greatest over the rapid growth phase of the tumour reaching a maximum 13 days after transplantation. This would suggest that the liver is influenced by growing tissue rather than necrotic tissue, for whilst the Walker tumour contains necrotic tissue at all stages of growth this would be greatest 21 days after transplantation. The

Fig. 4.—DEAE-cellulose chromatography of rat liver perchloric acid soluble protein. Unbound fraction was collected before commencement of gradient. For conditions of chromatography see text. Below is shown cellulose acetate electrophoresis of pooled fractions indicated by numbers above chromatogram.

findings of Macbeth, Bekesi and Tuba (1963) of a maximal elevation in total serum protein bound carbohydrate 14 days after Walker tumour implantation tends to support these results, although they did not measure the serum glycoprotein levels.

The increase in liver and spleen size during tumour growth has been reported many times previously (Sherman, Morton and Mider, 1950 ; Yeakel and Tobias, 1951 ; Stewart and Begg, 1953) and an increase both in nitrogen and in water content has been shown. The mechanism whereby the liver is induced to increase in size is not known although the work of Kampschmidt, Mayne, Goodwin and Clabaugh (1960), in which an increase in organ size was produced by injection of tumour extracts, indicates that it might be a humoral one. The proportionally greater increase in liver weight found here after 13 days' tumour growth suggests that the liver is being influenced by the growing tumour tissue. This is supported by the findings of Sherman *et al.* (1950) who found a greater increase in liver nitrogen content in rats bearing tumours 15–30 per cent of the total body weight than in rats bearing larger tumours.

The decrease in the acidic $HClO_4$ soluble components $G_R$ and $H_R$ appears to be related to the growth of the tumour, the effect being least in animals bearing slow-growing tumours. Sherman *et al.* (1950) have shown a rapid fall in the liver nitrogen level in tumour-bearing animals just prior to death and suggest that the animal is metabolising liver protein. The fall in the level of $G_R$ and $H_R$, takes place however, much earlier than the lethal phase of tumour growth and at a time when the other perchloric acid soluble proteins are elevated, so that it is possible that here also a humoral mechanism is involved.

SUMMARY

(1) Perchloric acid soluble proteins of rat liver contained $\alpha_1$-globulin and in addition several acidic and basic tissue components.

(2) The level of the liver $\alpha_1$-globulin was not altered in the tumour-bearing rat despite the large increases found in the serum glycoproteins.

(3) The tumour caused an increase in liver weight and in the total liver $HClO_4$ soluble protein which was maximal 13 days after implantation.

(4) Electrophoretic analysis revealed that the increase in $HClO_4$ soluble liver proteins was due mainly to an increase in the basic proteins whilst two acidic components $G_R$ and $H_R$ were decreased.

(5) Results are discussed in relation to the mechanism of glycoprotein synthesis.

We are grateful to the British Empire Cancer Campaign for Research and to the Endowment Funds of Westminster Hospital for generous financial support throughout this work and to Mr. B. C. V. Mitchley of the Chester Beatty Institute for Cancer Research for the gift of the Walker tumour. We thank Dr. J. Pryse-Davies for carrying out the histological examination of the tissues. We also thank Mrs. J. Rhodes for technical assistance.

### REFERENCES

BURSTON, D., APSEY, M. E., TOMBS, M. P. AND MACLAGAN, N. F.—(1963) in 'Protides of the Biological Fluids' Edited by H. Peeters. Proceedings of the 11th Colloquium, Bruges (1962) Elsevier Amsterdam 1962, p. 504.

*Idem*, TOMBS, M. P., APSEY, M. E. AND MACLAGAN, N. F.—(1963) *Brit. J. Cancer,* **17**, 162.

DARCY, D. A.—(1957) *Ibid.*, **11**, 137.
HOCHWALD, G. M., THORBECKE, G. J. AND ASOFSKY, R. T.—(1961) *J. exp. Med.*, **114**, 459.
KAMPSCHMIDT, R. F., MAYNE, M. A., GOODWIN, W. L. AND CLABAUGH, W. A.—(1960) *Cancer Res.*, **20**, 368.
KING, E. J. AND WOOTTON, I. D. P.—(1956) 'Microanalysis in Medical Biochemistry', London (Churchill) p. 35.
LOWRY, O. H., ROSEBOROUGH, N. J., FARR, A. L. AND RANDALL, A. J.—(1951) *J. biol. Chem.*, **193**, 265.
MACBETH, R. A. L., BEKESI, J. G. AND TUBA, J.—(1963) *Cancer Res.*, **23**, 938.
MILLER, L. L., HANAVAN, H. R., TITTHASIRI, N. AND CHOWDHURY, A.—(1964) *Advances in Chemistry Series*, **44**, 17.
PETERS, T.—(1962) *J. biol. Chem.*, **237**, 1181.
PETERSON, E. A. AND SOBER, H. A.—(1956) *J. Amer. chem. Soc.*, **78**, 751.
RICHMOND, J. E.—(1963) *Biochemistry*, **2**, 676.
ROBINSON, G. B., MOLNAR, J. AND WINZLER, R. J.—(1964) *J. biol. Chem.*, **239**, 1134.
SARCIONE, E. J.—(1962) *Biochemistry*, **1**, 1132.—(1963) *Arch. Biochem. Biophys.*, **100**, 516.
SHERMAN, C. D., MORTON, J. J. AND MIDER, G. B.—(1950) *Cancer Res.*, **10**, 374.
STEWART, A. G. AND BEGG, R. W.—(1953) *Ibid.*, **13**, 556.
TOMBS, M. P., COOKE, K. B., BURSTON, D. AND MACLAGAN, N. F.—(1961) *Biochem. J.* **80**, 284.
WEIMER, H. E., QUINN, F. A., REDLICH-MOSHIN, J. AND NISHIHARA, H.—(1957) *J. nat. Cancer Inst.*, **19**, 409.
YEAKEL, E. H. AND TOBIAS, G. L.—(1951) *Cancer Res.*, **11**, 830.